# EGFR Suppression Inhibits the Sphere Formation of MCF7 Cells Overexpressing EGFR

**DOI:** 10.32607/actanaturae.17857

**Published:** 2023

**Authors:** D. D. Novak, O. S. Troitskaya, A. A. Nushtaeva, M. V. Zhilnikova, V. A. Richter, M. I. Meschaninova, O. A. Koval

**Affiliations:** Institute of Chemical Biology and Fundamental Medicine, Siberian Branch of the Russian Academy of Sciences, Novosibirsk, 630090 Russian Federation; Department of Natural Sciences, Novosibirsk State University, Novosibirsk, 630090 Russian Federation

**Keywords:** 3D cell culture, spheroids, MCF7, EGFR, siRNA, cetuximab, AG1478

## Abstract

The epidermal growth factor receptor (EGFR) is an oncogenic tyrosine kinase that is involved in tumor initiation and progression, making EGFR inhibitors and monoclonal antibodies to this receptor essential for anti-tumor therapy. We have previously shown that EGFR transgene expression in the human breast adenocarcinoma cell line MCF7 (MCF7-EGFR) stimulates the 3D spheroid-like growth. The primary focus of our present work was to investigate whether EGFR inhibition could affect the assembly of spheroids or lead to the destruction of pre-existing spheroids. We compared the effects of anti-EGFR siRNA, the anti-EGFR monoclonal antibody cetuximab, and the tyrosine kinase inhibitor AG1478 on dissociated and spheroid MCF7-EGFR cells. MCF7-EGFR cells were found to have a 2.5-fold higher sensitivity towards the cytotoxic effects of cetuximab and AG1478 compared with the parental MCF7 cell line. The suppression of EGFR mRNA with siRNA was found to reduce the sphere formation, whereas treating the pre-existing spheroids had no such effect. Treatment of dissociated spheroids with cetuximab and AG1478 was also found to inhibit the MCF7-EGFR sphere formation. We suggest that EGFR expression is important, at least, during the spheroid formation stage. The transition of a MCF7wt adherent cell culture to MCF7-EGFR spheroids was accompanied by a considerable increase in N-cadherin adhesion proteins. The level of N-cadherin decreased when MCF7-EGFR cells were treated with siRNA and cetuximab. Thus, we have demonstrated that N-cadherin is involved in the EGFR-dependent formation of MCF7-EGFR spheroids. Accordingly, MCF7-EGFR spheroids can be considered a suitable model for studying aggressive hormone-positive breast tumors.

## INTRODUCTION

The interaction between various growth factors and their receptors is known to regulate the autonomous growth of cancer cells [1]. Hence, the epidermal growth factor (EGF) and its receptor (EGFR) play a crucial role in the pathogenesis and progression of various types of malignant tumors [2]. EGFR (or HER1) is a member of the ErbB family of receptor tyrosine kinases, which also includes HER2, HER3, and HER4. The EGFR composition includes an extracellular domain, a hydrophobic transmembrane domain, an intracellular catalytic tyrosine kinase domain, and several intracellular tyrosine residues [3].

Currently, two types of ErbB inhibitors are used in tumor therapy. There are monoclonal antibodies against the EGFR or HER2 extracellular domain, including cetuximab, matuzumab, panitumumumab, trastuzumab, and pertuzumab, as well as tyrosine kinase inhibitors that compete with ATP molecules for binding to the EGFR tyrosine kinase domain, such as gefitinib, erlotinib, lapatinib, AEE788 [4]. In 2004, the FDA first approved cetuximab for metastatic colorectal cancer, and in 2011, it was approved for head and neck cancer therapy [5, 6]. The competitive specific binding of cetuximab to EGFR was found to be effective in inhibiting receptor phosphorylation, which in turn impedes the EGFR signaling pathway and results in tumor cell proliferation [7].

Knockdown of therapeutically relevant target genes is also considered an effective strategy for tumor therapy. Inhibition of mRNA processing by small interfering RNA (siRNA) is regarded as one way to block a specific target. RNA interference is a protective mechanism against exogenous nucleic acids entering the cell, such as viral RNA [8]. Currently, preclinical and clinical trials are under way on several siRNA-based agents for the treatment of brain and prostate cancer [9].

Earlier, we obtained an MCF7 human breast adenocarcinoma cell line that manifested an increased expression of EGFR. The study revealed that excessive EGFR in MCF7 cells led to the spontaneous formation of spheres under standard culture conditions [10, 11]. MCF7-EGFR spheroids have a round shape with a well-defined outer boundary and a median diameter of 100 μm, with the size of large spheroids likely to exceed 400 μm. Given that EGFR production has been demonstrated to affect the adhesive properties of MCF7-EGFR cells, inhibition of EGFR may be believed to cause disruption of the formed spheroids or inhibit the assembly of spheroids from individual cells. To verify this hypothesis, we examined the effects of anti-EGFR siRNA, cetuximab, and the tyrosine kinase inhibitor AG1478 on the structure and formation of MCF7-EGFR spheroids.

## EXPERIMENTAL PART

**Cell lines**

The human breast adenocarcinoma cell lines MCF7wt (#ACC 115, Germany) and MDA-MB-231 (#ACC 732, Germany) were investigated. The cells were cultured as a monolayer in a IMDM or DMEM medium, containing 10% FBS and 1% penicillin-streptomycin-amphotericin (hereafter, complete medium), respectively, as described earlier [12].

The human breast adenocarcinoma cell line MCF7-EGFR forming spheroids was described in the previous study [10]. MCF7-EGFR spheroids were cultured under standard conditions on non-adhesivecoated plates (Nest Bio-technology Co., China).

**Spheroid formation and counting**

For spheroid formation kinetic curves to be generated, the cells were dissociated using Stempro™ Accutase™ reagent (Gibco, USA), seeded at 3 × 10^4^ cells/well into a 48-well, non-adhesive-coated plate (Eppendorf, Germany), and cultured under standard conditions, as described above. The spheroids were counted in three or six independent wells of the plate using an inverted microscope (Eclipse Ti, Nikon, Japan) at 40× magnification. All free-floating spheroids larger than 30 μm were counted in the light field. Then, the average number of spheroids per well and the standard deviation (SD) were calculated. Preliminary counts in all experiments were performed using the ImageJ software (version 1.52a, USA) (data not shown). The exact number of spheroids was calculated manually.

**Anti-EGFR siRNA construction**

To evaluate the EGFR inhibition on MCF7-EGFR spheroids, we constructed siRNAs based on the sequences described in [13]. The oligonucleotides were synthesized in the laboratory of RNA chemistry of the Institute of Chemical Biology and Fundamental Chemistry of the Siberian Branch of the Russian Academy of Sciences. We used the following siRNAs: senScr 5’-CAA GUC UCG UAU GUA GUG GUU-3’, antiScr 5’-CCA CUA UAU ACG AGA CUU GUU-3’, senEGFR 5’-GUC CGC AAG UGU AAG AAG UTT-3’, antiEGFR 5’-ACU UCU UACU ACU UGC GGA CTT-3’. The average concentration of ribooligonucleotides in the solution was calculated to be 0.203 mM.

**siRNA hybridization**

Equimolar amounts of sen- and anti-sen siRNAs were mixed with 5-fold siRNA hybridization buffer (100 mM C2H3NaO_2_, 30 mM HEPES-KOH, 2 mM Mg(CH_3_COO)2, pH 7.4) in the ratio 2 : 2 : 1. The samples were heated in a water bath for 2 min at 90°C and cooled to room temperature. Two volumes of 1× siRNA hybridization buffer were added to the resulting mixture. The final concentration of siRNA duplexes was 27 μM.

**siRNA cell transfection**

The siRNA transfection was performed using Lipofectamine 3000 reagent (Invitrogen, USA) according to the manufacturer’s protocol. The cells were plated 24 h before the experiment, treated with 100 nM siRNA, and incubated for 4 h at 37°C. Then, the medium was replaced with a complete medium suitable for the cell culture and the cultivation was continued.

**Cell survival assay**

The cell viability was determined 72 h after treatment with the drug using the MTT test as described in [12]. The IC_50_ values were calculated using the CompuSyn version 1.0 software. The initial solution   (5 mg/ml) of cetuximab Erbitux® (Merck Healthcare, Germany) was stored at +4°C. For cell culture experiments, cetuximab was diluted in a complete IMDM medium. The stock solution (31.7 mM) of AG1478 (Sigma-Aldrich, USA) in DMSO:MeOH (1 : 1) was stored at -20°C. For cell culture experiments, AG1478 was diluted in a complete IMDM medium so that the DMSO concentration in the wells was 0.5%.

**FDA staining**

An initial solution (1 mg/ml) of fluorescein diacetate, FDA (Sigma-Aldrich, USA), diluted in DMSO was stored at -20°C. The solution was added to the culture medium until the final concentration of 10 μg/ml. The spheroids were incubated in a complete IMDM medium with dissolved FDA for 30 min, followed by harvesting with centrifugation and washing with PBS. A fluorescence microscope (Eclipse Ti, Japan) and flow cytometry were used to analyze the cell viability and cytotoxicity.

**Flow cytometry**

Following the treatment, the spheroids were dissociated with Stempro™ Accutase™ reagent, washed in PBS, and incubated with antibodies to EGFR to determine EGFR levels. The cells were incubated with propidium iodide (PI) or FDA according to the manufacturer’s protocol for viability assay. We used the following antibodies: mouse IgG monoclonal antibodies to the EGFR protein (Invitrogen, USA), secondary antibodies conjugated with the Alexa Fluor 647 fluorescent tag (Abcam, UK). All the assays were performed using a FACSCantoII flow cytometer (BD Biosciences, USA). The data were analyzed using the FACSDiva software (BD Biosciences, USA). The cell populations were isolated using forward and side light scattering to exclude small particles. At least 10,000 events were collected in each experiment.

**Western blot analysis**

Western blot analysis was performed according to the protocol described in [14]. The cells were lysed, the protein concentration was measured, and then the samples (15 μg) were separated using 10% SDS-PAGE and transferred to a PVDF membrane. The membrane was blocked with a 5% milk powder solution and incubated sequentially with primary and secondary antibodies conjugated with horseradish peroxidase. We used the following antibodies: primary IgG antibodies to actin (Sigma-Aldrich, USA), EGFR (Santa Cruz Biotechnology, USA), SNAIL + SLUG (Abcam), N-cadherin (Invitrogen, USA), E-cadherin (Abcam), and horseradish peroxidase conjugates of secondary antibodies to rabbit (Thermo Fisher, USA) and mouse antigens (Thermo Fisher). The chemiluminescent signal was recorded using the Novex ECL HRP reagent kit (Invitrogen) and the GE Amersham Imager 600 (GE, USA). Densitometric analysis of Western blots was performed using the GelAnalyser version 2010a image analysis software.

**Statistical analysis**

The results are presented as the arithmetic mean ± SD for the sample. Statistical analysis was performed using Student’s *t*-criterion. The differences were considered statistically significant at *p* < 0.05.

## RESULTS AND DISCUSSION

**Effect of anti-EGFR siRNA on MCF7-EGFR spheroids**

We evaluated the effect of EGFR downregulation on MCF7-EGFR spheroid formation by anti-EGFR siRNA. An international database of NCBI Nucleotides was used to determine the complementarity of the selected anti-EGFR siRNA to the sequence of exon 8 of the human EGFR gene. This exon encodes a fragment of EGFR subdomain III responsible for receptor-ligand binding [15].

**Fig. 1:**
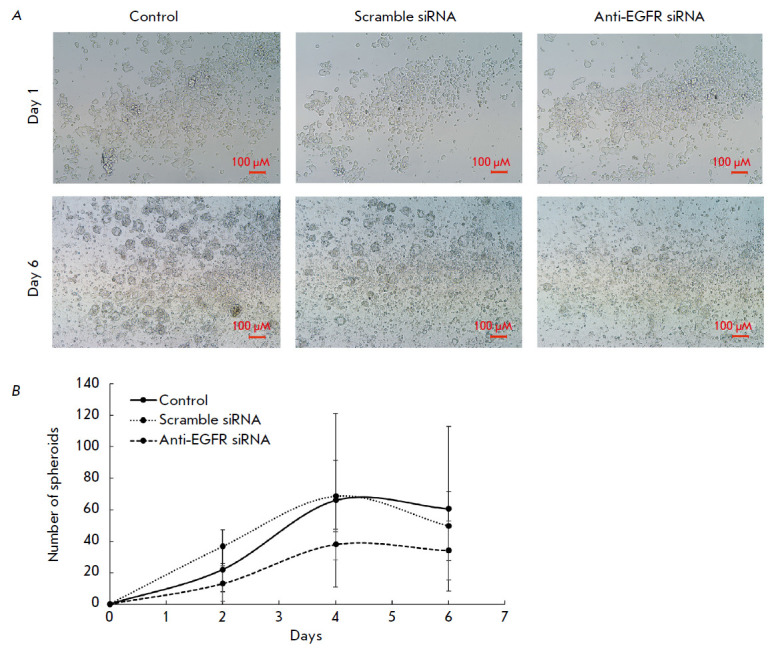
Effect of anti-EGFR siRNA on the formation of MCF7-EGFR spheroids. (*A*) the photographs of the control and siRNA-treated MCF7-EGFR spheroids. (*B*) the growth dynamics of MCF7-EGFR spheroids. The dissociated spheroids were seeded into 48- well plates, treated with siRNA (100 nM), and counted in separate wells, with the number of spheroids reported per well. The control cells were treated with LF

The MCF7-EGFR spheroids were dissociated into individual cells and then seeded into plates with 100 nM anti-EGFR siRNA. Lipofectamine 3000 (LF) was used as a transfection agent. Scrambled siRNA was used as a negative control, and cells treated only with LF were used as a control for the cytotoxic activity of LF. The dynamics of growth and sphere formation after siRNA treatment and in the control samples were evaluated by automatic and direct sphere counting. Treatment of the cells with anti-EGFR siRNA resulted in a reduction in the number of spheroids compared to the control cells and the cells treated with Scramble siRNA (*Fig. 1 A,B*).

**Fig. 2:**
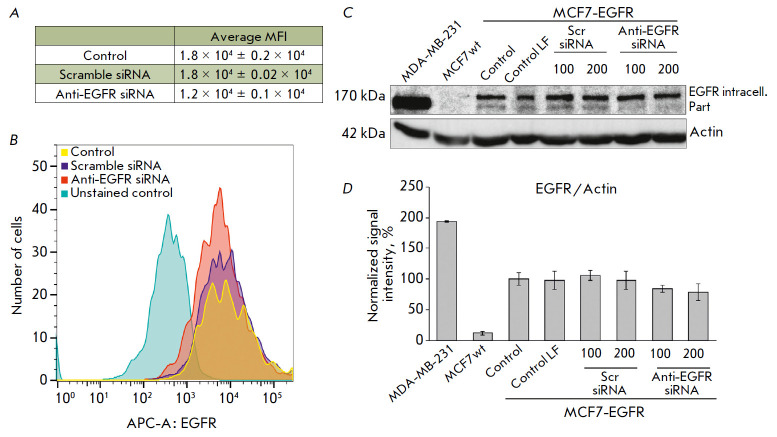
Evaluation of EGFR reduction in MCF7-EGFR cells under the action of anti-EGFR siRNA. (*A*) the quantification of the EGFR+ cell population. The data are presented as the mean fluorescence intensity (MFI) of EGFR + cells relative to the control cells ± SD from two independent experiments. (*B*) the representative image of the cytometric analysis. (*C*, *D*) the changes in EGFR levels after anti-EGFR siRNA treatment. MDA-MB-231, MCF7wt were used as control cell lines. The MCF7-EGFR spheroids were dissociated and treated with Scramble siRNA, anti-EGFR siRNA (100–200 nM) for 48 h. (*C*) the representative images of the Western blot analysis. (*D*) the Western blot analysis of EGFR/actin in the cells

The level of total cellular EGFR in MCF7-EGFR cells was found to be almost 10-fold higher than that in the MCF7wt cells (*Fig. 2 B,D*). The EGFR knockdown caused by anti-EGFR siRNA was assessed by flow cytometry and Western blotting using antibodies to the surface and internal domains of the protein, respectively. The decrease in the level of surface EGFR on the second day after treatment of the MCF7-EGFR spheroids with siRNA was estimated to be 20–25% (*Fig. 2*). The Western blotting data are consistent with the results of a measuring of the surface EGFR levels in the cells treated with siRNA.

**Fig. 3:**
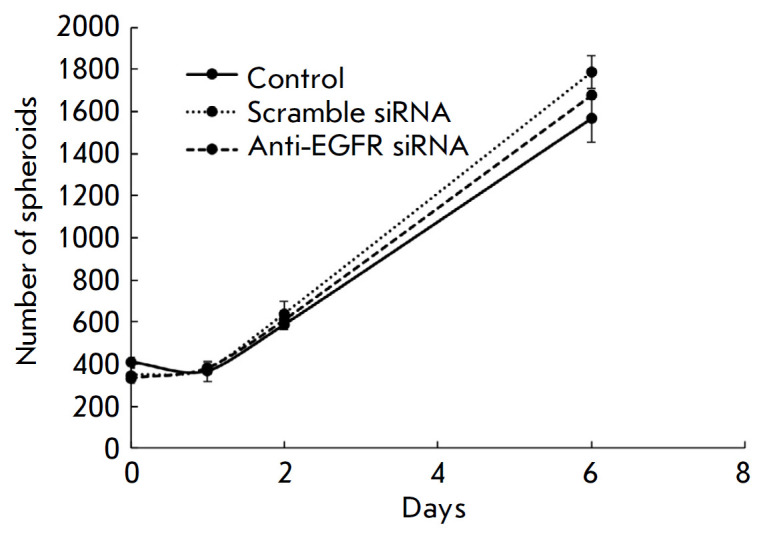
Analysis of the effect of anti-EGFR siRNA on the structure of MCF7-EGFR spheroids. The growth kinetics of MCF7-EGFR spheroids. The spheroids were seeded, treated with siRNA (100 nM), and counted in separate wells of 24-well plates with the number of spheroids per well. The control spheroids were treated with LF. The data are presented as the mean ± SD of three independent experiments

The influence of anti-EGFR siRNA on the formed structures was detected by placing the MCF7-EGFR spheroids in a non-adhesive plate and following incubation with siRNA. Anti-EGFR siRNA was found to have no effect on the spheroid structure (*Fig. 3*). siRNAs at a concentration of 20–200 nM are commonly used to effectively inhibit target protein expression [16, 17, 18]. However, the siRNA penetration into the spheroids may proceed worse than in the cells growing in a monolayer. Therefore, higher concentrations of siRNA are often used in experiments with spheroids or transfection when performed in a serum-supplemented medium [19, 20]. It is noteworthy that, in our study, increasing the concentration of anti-EGFR siRNA to 200 nM did not lead to a further decrease in EGFR levels. The transfection in the serum medium did not increase the efficiency of EGFR suppression in the MCF7-EGFR spheroids (data not shown). We believe further optimization of MCF7-EGFR spheroid transfection with siRNA to be highly relevant.

The data obtained suggest that suppression of EGFR by specific siRNAs at the spheroid assembly stage leads to a decrease in the rate of spheroid formation in a MCF7-EGFR culture. At the same time, suppressing EGFR in mature spheroids does not lead to their destruction.

**Effect of cetuximab on MCF7wt cells and mature MCF7-EGFR spheroids**

**Fig. 4:**
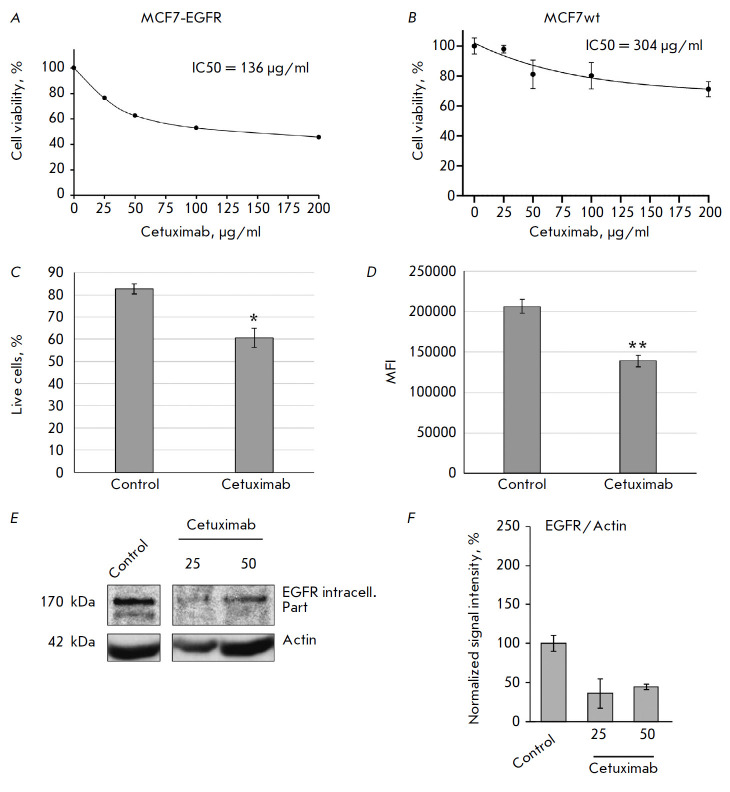
The cytotoxic activity of cetuximab against MCF7-EGFR spheroids (*A*) and wild-type MCF7 cells (*B*). The IC_50_ values were determined by the MTT assay. (*C*, *D*) – the estimation of the proportion of live MCF7-EGFR spheroid cells after cetuximab treatment by flow cytometry. The MCF7-EGFR cells were incubated with cetuximab (50 μg/ml) for 72 h and stained with FDA. (*C*) the mean % of live cells ± SD of two independent experiments. (*D*) MFI, the mean fluorescence intensity of live cells. The differences were significant at *p  < 0.05, ** *p* < 0.01. (*E*, *F*) the changes in the EGFR levels after cetuximab treatment. MCF7-EGFR spheroids were dissociated and treated with cetuximab (25–0 μg/mL) for 48 h. (*E*) the representative images of the Western blot analysis. (*G*) the Western blot analysis of EGFR/actin in the cells

Given that cetuximab binding to the target causes cell death, this drug is used in the immunotherapy of EGFR-positive malignancies [21]. We evaluated the cytotoxic activity of cetuximab against MCF7-EGFR spheroids: the drug (25–200 µg/ml) was added to the spheroids as they were left to continue to cultivate under standard conditions for 72 h. The cells were then stained with propidium iodide (PI), and the percentage of PI-negative cells was determined by flow cytometry, corresponding to the population of living cells (*Fig. 4A*). The IC_50_ value of cetuximab was 136 μg/mL for the MCF7-EGFR spheroids and 304 μg/mL for the MCF7wt parental cell line, which was 2.5-fold higher than that for the MCF7-EGFR spheroid cells, indicating cell resistance to the drug (*Fig. 4B*). By comparing the experimental IC_50_ values with published data for other EGFR-positive tumor cells, such as lung cancer A549 (IC_50_ = 146 μg/ml) [22], the MCF7-EGFR cell line can be characterized as cetuximab-sensitive. Thus, MCF7-EGFR spheroid cells cultured under standard conditions can be treated with cetuximab.

Since the study aimed to assess the non-cytotoxic effects of cetuximab on spheroids, cetuximab concentrations lower than the IC25 value were further used. Fluorescein diacetate (FDA), an esterase substrate capable of penetrating the cell, was used to visualize the living cells. FDA can be used as a viability assay tool that measures both enzymatic activity and cell membrane integrity [23]. The MCF7-EGFR spheroids were treated with cetuximab (50 μg/ml) for 72 h and stained with FDA. Flow cytometry of the cells treated with cetuximab revealed a decrease in the population of live cells by up to 20% of the values in the control group (*Fig. 4 B,D*). The samples of spheroids not treated with the cytotoxic agent also had dead cells, the presence of which can be explained by the formation of necrotic spheroid nuclei caused by the lack of oxygen and nutrient transport, as we described earlier [10]. Cetuximab was found to decrease the EGFR level more considerably than anti-EGFR siRNA: up to 60% relative to untreated MCF7-EGFR cells (*Fig. 4 D,E*).

**Adding cetuximab at the stage of dissociated spheroid cells reduces MCF7-EGFR spheroid formation**

**Fig. 5:**
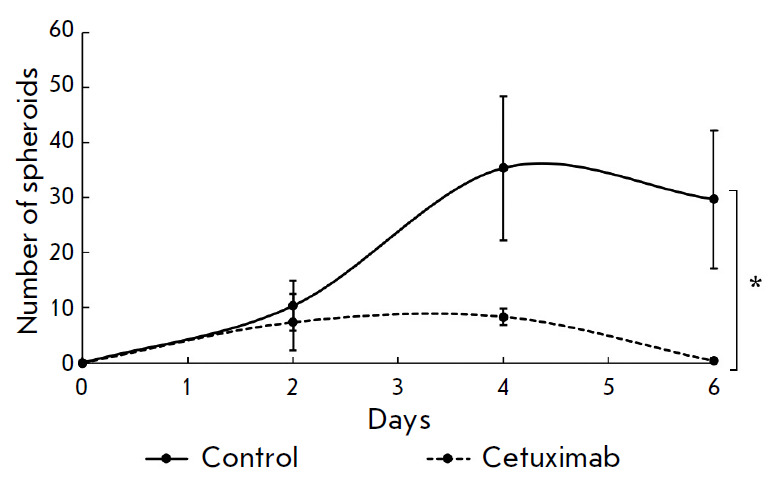
Analysis of the cetuximab effect on MCF7-EGFR spheroid formation. The spheroids were dissociated, seeded in a 48-well nonadhesive plate, treated with cetuximab (50 μg/ml), and counted in individual wells. The data are presented as the mean ± SD of three independent experiments; *p  < 0.05

The dynamics of spheroid formation was assessed after treatment with cetuximab to confirm the ability of cetuximab to inhibit the formation of MCF7-EGFR spheroids. MCF7-EGFR spheroids were dissociated and cultured under standard conditions in the presence of cetuximab (50 μg/ml). The cetuximab treatment resulted in complete suppression of sphere formation on the sixth day of cultivation (*Fig. 5*).

**Fig. 6:**
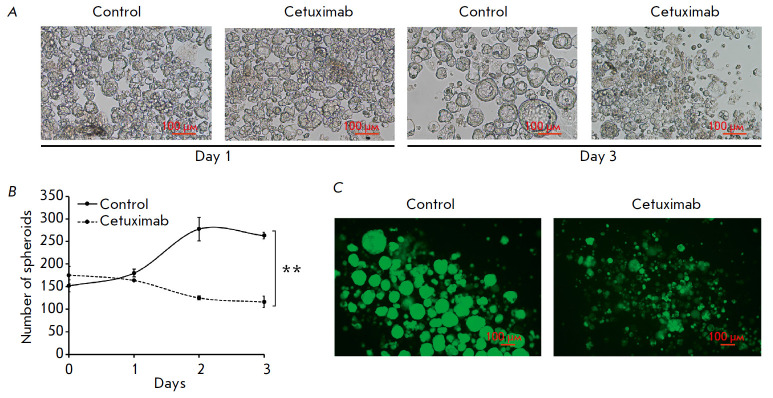
Analysis of the cetuximab effect on the structure of the MCF7-EGFR spheroids. (*A*) the photographs of the control and cetuximab-treated MCF7-EGFR spheroids. (*B*) the growth kinetics of the MCF7-EGFR spheroids cultured in a medium with and without cetuximab. The spheroids were seeded, treated with cetuximab (50 μg/ml), and counted in the individual wells of 48-well plates, and the number of spheroids per well was indicated. The data are presented as the mean value ± SD of three independent experiments; **p  < 0.01. (*C*) The microscopic analysis of MCF7-EGFR spheroids treated with cetuximab for 72h and stained with FDA

In addition, we evaluated the effect of cetuximab on the spheroids formed. The MCF7-EGFR spheroids were seeded on a non-adhesion culture plate in a cetuximab-added medium (50 μg/ml) for 72 h. The cetuximab treatment resulted in a decrease in the number of spheroids, indicating that MCF7-EGFR spheroid degradation was stimulated when EGFR was inhibited (*Fig. 6 A,B*). The spheroids treated with cetuximab and stained with FDA were analyzed by fluorescence microscopy. Multiple individual cells appeared in the presence of cetuximab, with the number of large and structured spheroids decreasing compared with the control (*Fig. 6B*).

Following the exposure to the cetuximab inhibitor, the number of spheroids decreased compared to untreated samples, with this effect shown for both dissociated spheroids and already-formed spheroids. We believe cetuximab to have a predominantly persistent antiproliferative effect on both new and existing spheroids. In contrast to siRNA, the efficacy of cetuximab delivery to the cells of the inner layer of the spheroid is not in doubt since the effects of cetuximab have been confirmed at the organismal level [5].

**Effect of AG1478 on the MCF7-EGFR cell spheroid formation**

**Fig. 7:**
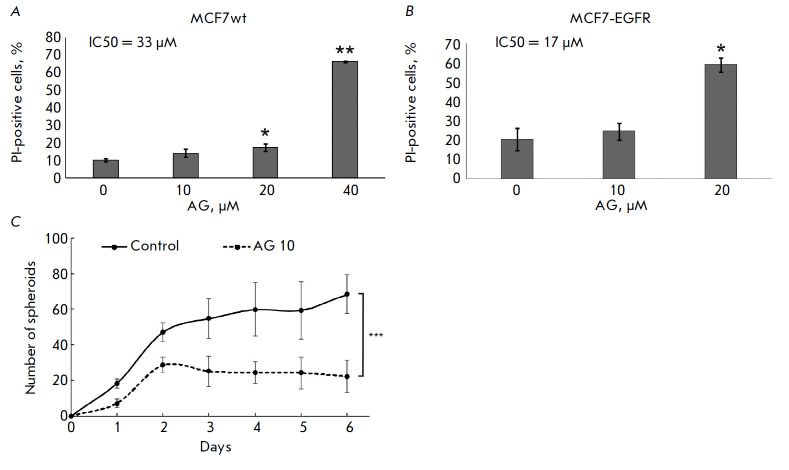
The cytotoxic activity of the EGFR inhibitor AG1478 (AG) against wild-type MCF7 cells. (*A*) and MCF7-EGFR spheroids (*B*). MCF7wt cells or MCF7-EGFR spheroids were incubated with AG (10–40 μM) for 5 days. The control cells were incubated with DMSO. After the treatment, the spheroids were dissociated, stained with propidium iodide (PI), and analyzed by flow cytometry. Data presented as the mean % of PI-positive cells ± SD of two independent experiments. (*C*) Growth curves of the MCF7-EGFR spheroids. The distorted spheroids were seeded in 48-well plates, and after 24 h of incubation the cells were treated with AG (10 μM). The spheroids were counted in individual wells. The data are presented as the mean value ± SD of six independent experiments, with *p  < 0.05, **p  < 0.01, ***p < 0.001

We analyzed how the MCF7-EGFR cells were affected by the EGFR inhibitor tyrphostin (AG1478, or AG), known to inhibit the binding of ATP molecules to the intracellular domain of the receptor. The IC_50_ value of AG1478 for wild-type MCF7 cells was determined to be almost twice as high as that for MCF7-EGFR cells (*Fig. 7 A,B*). Thus, we have demonstrated a change in the sensitivity of MCF7-EGFR cells to EGFR-inhibitory agents compared to MCF7wt cells.

To analyze the effect of AG1478 on spheroid formation, we dissociated MCF7-EGFR spheroids, seeded them onto plates, and added AG1478 (10 μM) to the cells after 24 h of cultivation. The AG1478 treatment significantly reduced the number of spheroids compared to the control cells (*Fig. 7B*).

**Effect of siRNA and cetuximab on the levels of adhesion proteins and epithelialmesenchymal transition regulatory proteins**

Rao et al. have demonstrated that EGFR regulates the integrin activation and the spatial organization of focal adhesions [24]. Therefore, it is worth studying the relationship between EGFR levels and spheroid formation when not only horizontal, but also vertical interactions are formed between cells and significant changes in the adhesion properties occur. Cell adhesion is considered to be an important component controlling the interactions between cells and their environment. EGFR has been shown to destabilize E-cadherin-mediated adhesion by enhancing E-cadherin endocytosis, modifying its interaction with the cytoskeleton, and reducing its expression, thereby promoting oncogenesis [25]. To compare the effects of anti-EGFR agents on the levels of certain proteins in MCF7-EGFR spheroids treated with siRNA or cetuximab and in parental MCF7wt cells, we analyzed the levels of SNAIL/SLUG, N-cadherin, and E-cadherin by Western blotting.

**Fig. 8:**
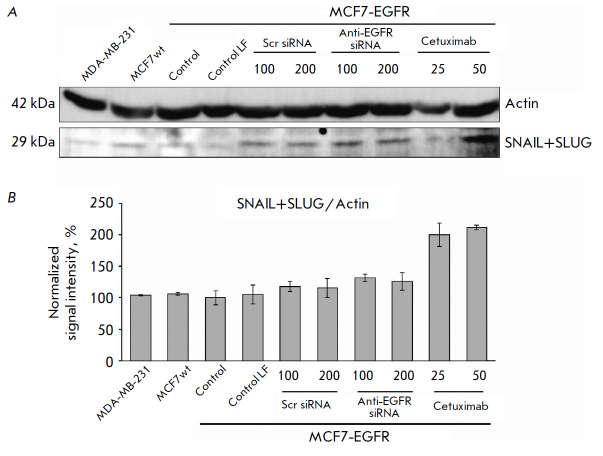
The changes in cellular proteins after the anti-EGFR siRNA and cetuximab treatment. MDA-MB-231, MCF7wt were used as control cell lines. The MCF7-EGFR spheroids were dissociated and treated with Scramble siRNA, anti-EGFR siRNA (100–200 nM), or cetuximab (25–50 µg/ml) for 48 h. (A) the representative pictures of the Western blots analysis. (B) the Western blots analysis of SNAIL+SLUG/Actin in the cells

The transcription factors SNAIL and SLUG were reported to be involved in the regulation of epithelial- mesenchymal transition, an important factor in three-dimensional models [26]. We found no differences in the basal level of SNAIL/SLUG proteins between the MCF7wt and MCF7-EGFR cell lines. Although the incubation with siRNA had no effect on the SNAIL/SLUG levels, cetuximab treatment resulted in a twofold increase in the SNAIL/SLUG levels in the MCF7-EGFR cells (*Fig. 8 A,B*).

**Fig. 9:**
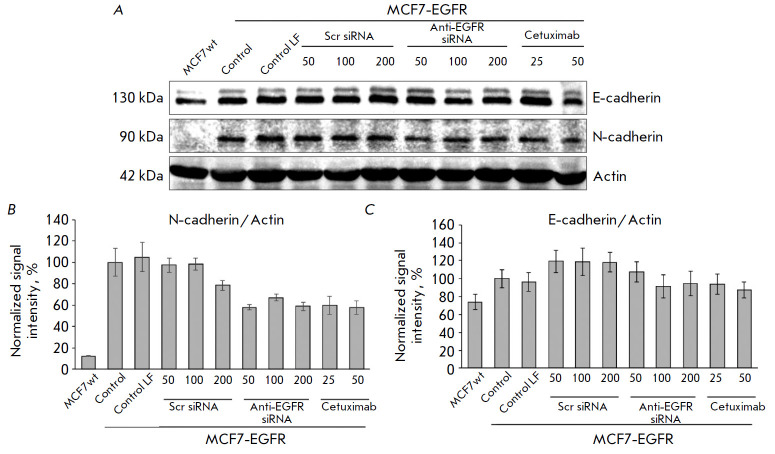
The changes in cellular proteins after anti-EGFR siRNA and cetuximab treatment. MDA-MB-231, MCF7wt were used as control cell lines. The MCF7-EGFR spheroids were dissociated with accutase and treated with Scramble siRNA (scr siRNA), anti-EGFR siRNA (50–200 nM), or cetuximab (25–50 μg/ml) for 48 h. (*A*) The representative pictures of the Western blots analysis. (*B*, *C*) the Western blots analysis of N-cadherin/Actin (*B*) and E-cadherin/Actin (*C*)

The N-cadherin to E-cadherin ratio in the cell is considered to be an important factor determining intercellular adhesion and spheroid formation [27]. We have found the baseline level of N-cadherin in the MCF7-EGFR cell line to be more than 5-fold higher than that in the original MCF7wt cell line. The anti- EGFR agent treatment led to a decrease in the level of N-cadherin in MCF7-EGFR spheroids (*Fig. 9 A,B*). The baseline level of E-cadherin in MCF7-EGFR cells did not differ statistically significantly from that in the MCF7-EGFR spheroids. Moreover, the level of E-cadherin in the MCF7-EGFR spheroids was not affected by the addition of anti-EGFR siRNA or cetuximab (*Fig. 9 A,C*).

## CONCLUSIONS

We have revealed the tyrosine kinase receptor EGFR to be involved in the maintenance of MCF7-EGFR cell sphere formation. The importance of EGFR in spheroid formation was confirmed in experiments with EGFR inhibitors. The suppression of EGFR at the stage of individual cells has been demonstrated to reduce spheroid formation, whereas the treatment of already-formed spheroids showed no such effect. We suggest the EGFR expression to be significant, at least at the stage of spheroid formation. For a significant knockdown effect of the EGFR gene using siRNA on large spheroids, the efficiency of the transfection system should be increased. In addition, we have demonstrated the transition of MCF7-EGFR cells into three-dimensional structures to be associated with a significant increase in the expression of the N-cadherin protein. Our findings led us to assume that sphere formation by MCF7-EGFR cells could be partially related to the cellular pathways regulating the epithelial-mesenchymal transition. The results obtained in this study appear to owe in part to the properties of MCF7 cells, with high-EGFR MDA-MB-231 cells not forming spheroids without the addition of growth factors and matrices. Nevertheless, developing such a cell model with abnormal N-cadherin activation is essential for identifying potential molecular targets of tumor progression. Moreover, MCF7-EGFR spheroids are considered to be a model for testing the therapeutic effects of the combination of EGFR and N-cadherin inhibitors.

## Abbreviations

EGFR: epidermal growth factor receptor
siRNA: small interfering RNA
LF: Lipofectamine 3000
PI: propidium iodide
wt: wild type
AG: EGFR inhibitor (AG1478)
DMSO: dimethyl sulfoxide
FDA: fluorescein diacetate
SD: standard deviation
IC50: drug concentration, at which cell death reaches 50%
